# Early brain injury and cognitive impairment after aneurysmal subarachnoid haemorrhage

**DOI:** 10.1038/s41598-021-02539-x

**Published:** 2021-12-01

**Authors:** Matthew J. Rowland, Payashi Garry, Martyn Ezra, Rufus Corkill, Ian Baker, Peter Jezzard, Jon Westbrook, Gwenaëlle Douaud, Kyle T. S. Pattinson

**Affiliations:** 1 Nuffield Division of Anaesthetics, Nuffield Department of Clinical Neurosciences, University of Oxford, Oxford, OX3 9DU UK; 2 Neurosciences Intensive Care Unit, Oxford University Hospitals NHS Trust, Oxford, UK; 3 Department of Psychological Medicine, Oxford University Hospitals NHS Foundation Trust, Oxford, OX3 9DU, UK; 4 FMRIB, Wellcome Centre for Integrative Neuroimaging, Nuffield Department of Clinical Neurosciences, University of Oxford, Oxford, UK

**Keywords:** Prognostic markers, Stroke, Diagnostic markers

## Abstract

The first 72 h following aneurysm rupture play a key role in determining clinical and cognitive outcomes after subarachnoid haemorrhage (SAH). Yet, very little is known about the impact of so called “early brain injury” on patents with clinically good grade SAH (as defined as World Federation of Neurosurgeons Grade 1 and 2). 27 patients with good grade SAH underwent MRI scanning were prospectively recruited at three time-points after SAH: within the first 72 h (acute phase), at 5–10 days and at 3 months. Patients underwent additional, comprehensive cognitive assessment 3 months post-SAH. 27 paired healthy controls were also recruited for comparison. In the first 72 h post-SAH, patients had significantly higher global and regional brain volume than controls. This change was accompanied by restricted water diffusion in patients. Persisting abnormalities in the volume of the posterior cerebellum at 3 months post-SAH were present to those patients with worse cognitive outcome. When using this residual abnormal brain area as a region of interest in the acute-phase scans, we could predict with an accuracy of 84% (sensitivity 82%, specificity 86%) which patients would develop cognitive impairment 3 months later, despite initially appearing clinically indistinguishable from those making full recovery. In an exploratory sample of good clinical grade SAH patients compared to healthy controls, we identified a region of the posterior cerebellum for which acute changes on MRI were associated with cognitive impairment. Whilst further investigation will be required to confirm causality, use of this finding as a risk stratification biomarker is promising.

## Introduction

Aneurysmal subarachnoid haemorrhage (SAH) is a devastating disease with high mortality and morbidity in those patients who survive hospital treatment. Despite accounting for only 5% of all strokes, the socioeconomic cost of SAH has remained disproportionately high with a loss of functional years equivalent to ischaemic stroke. This is because despite reductions in the incidence of SAH^1,2^ and mortality^3^, the disease often affects young adults of working age and frequently results in poor neurocognitive outcome^4^.

Following successful aneurysm treatment, the management of patients with SAH is conventionally focused on the prevention, diagnosis and treatment of secondary brain injury caused by delayed cerebral ischaemia^5^. However, there is increasing evidence that damage caused in the first 72 h post-rupture—so called “early brain injury”—may play a key role not only in the development of such delayed cerebral ischaemia, but also in determining overall neurocognitive outcomes^6^. Early cerebral ischemia and infarction is common in the acute period after SAH and is associated with worse neurological and physiological admission status, as well as poor neurocognitive outcomes^7,8^. Global cerebral oedema is also a significant feature of early brain injury and is an independent risk factor for mortality and poor outcome after SAH^9,10^.

Advances in MRI physics and analysis now offer a number of objective, reliable and non-invasive tools such as apparent diffusion coefficient (ADC) and voxel-based morphometry (VBM) to measure changes in cerebral structure at a tissue level. The ADC is a measure of the diffusivity of water molecules in tissue. In ischemic tissue e.g. following SAH, cytotoxic edema leads to a reduction in the ADC suggesting that the ADC may be a good parameter for identification/quantification of cerebral edema^11–13^. Some of the main advantages to using a voxel-by-voxel approach such as VBM are that firstly, it is not biased to one particular structure, and gives a comprehensive assessment of anatomical differences throughout the entire brain without a priori knowledge of which regions may be affected. Furthermore, it is a fully automated process, and therefore is fully reproducible intra- and inter-rater^14,15^. For this reason, it is highly suited to the investigation of early brain injury due to the unpredictable nature of the physiological insult that occurs following aneurysm rupture. Furthermore, as early brain injury is characterised by cerebral oedema, using imaging techniques such as ADC and VBM that are sensitive to regional changes in brain tissue is particularly of value.

The primary aim of this study was therefore to characterise imaging biomarkers of early brain injury in patients with good clinical grade SAH (WFNS 1 and 2). We aimed to quantify global and regional grey matter volume and apparent diffusion coefficient (ADC) in patients at three time points: within the first 72 h post-rupture, at 5–10 days and at 3 months post-SAH. We then investigated whether we could predict, within the acute period of the first 72 h post-SAH, the future incidence of cognitive impairment at 3 months post-SAH. Finally, we conducted an exploratory analysis relating our imaging findings with those from the UK Biobank to investigate possible genetic mechanisms underpinning any anatomical differences observed in those with poor cognitive outcome. Our study aimed to build a greater understanding of the pathophysiological changes incurred by aneurysm rupture, and to identify which patients—amongst those initially faring better—were most at risk of poor cognitive outcomes.

## Results

### Clinical demographics

**Figure 1:**
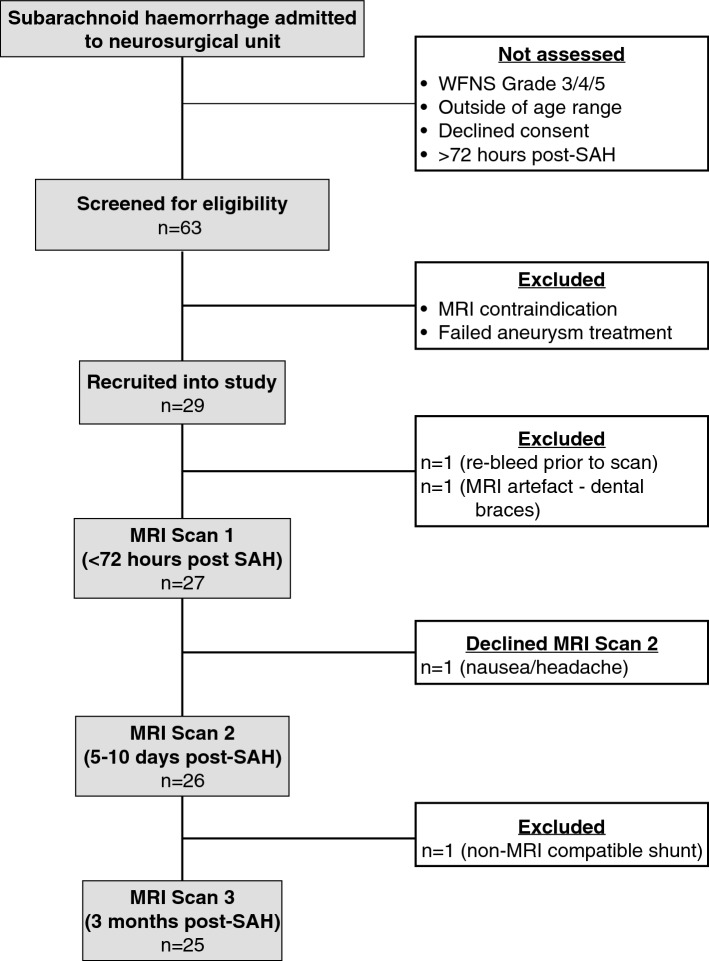
CONSORT diagram of study recruitment.

Between March 2011 and May 2015, 63 patients were screened for eligibility into the study. Of these, 29 matched inclusion criteria and were recruited (Table 1). Complete demographics and radiological data for each recruited patient are included in the Supplemental Material (Table S2). Figure 1 shows the CONSORT diagram outlining the recruitment and progress of the study. 27 healthy control subjects, paired by age (± 2 years) and gender to the recruited patients who underwent successful MRI scanning (n = 27) were also recruited.

**Table.1:** Demographics of the scanned patients and controls (*2 patients did not undergo cognitive testing).

Overall demographics	All patients	Controls	Impaired*	Non-impaired*
n	27	27	11	14
Mean age (range)	55 (31–77)	55 (32–77)	53 (37–72)	55 (31–70)
Gender (M:F)	11:16	11:16	4:7	5:9
Premorbid IQ: mean NART (std)	104 (12)		99 (2)	106 (3)
**WFNS grade**				
Median	1		1	1.5
1	17		10	7
2	10		1	7
**Modified Fisher grade**				
Median	4		4	4
1	0		0	0
2	0		0	0
3	6		3	4
4	21		8	10
**Aneurysm location**				
Anterior circulation	12		5	6
Middle cerebral artery	6		0	2
Internal carotid artery	8		3	2
Posterior circulation	1		3	4
Left side	12		4	7
Right side	13		6	6
Midline	2		1	1
**Complications**				
Hydrocephalus	13		4	8
Extra-ventricular drain	2		0	1
DCI/DCI-related cerebral infarction	8		3	5
Angiographic vasoconstriction (measured on CT angiogram)	1		0	1
**Mean time post-SAH to assessment (range)**				
Assessment 1	52 h (29–65)			
Assessment 2	6 days (5–10)			
Assessment 3	84 days (74–108)

### Cognitive outcomes

Of the 25 patients who attended and completed formal cognitive testing at Assessment 3 (3 months post-SAH), 11 patients were diagnosed as being cognitively impaired based on their N-ISAT scores. Out of the 5 cognitive domains investigated, patients with impairment were specifically worse in their executive skills, processing speed and non-verbal skills (Supplementary Material—Fig. S1). Those cognitively impaired patients based on lower N-ISAT scores also had significantly lower ACE-R scores than those with no impairment (89 ± 5 vs. 93 ± 5, p = 0.03 Mann–Whitney test). There were no significant differences between the impaired and non-impaired groups in age (p = 0.84), gender (p = > 0.99) and Fisher score (p = 0.85) (Table 1).

Using the NART to estimate pre-morbid IQ, there were also no differences between impaired and non-impaired patients (Table 1). However, post-SAH IQ at Assessment 3 using the WASI showed a significant decrease in IQ in those patients with cognitive impairment compared with those without.

### MRI results

Complete T1-weighted data was obtained for 27 patients (Fig. 1), while diffusion-weighted data was obtained in 14 patients due to constraints on length of time in the scanner for some patients.

#### Cross-sectional group comparison analyses (patients vs. controls)

##### Assessment 1: < 72 h post-SAH

**Figure 2:**
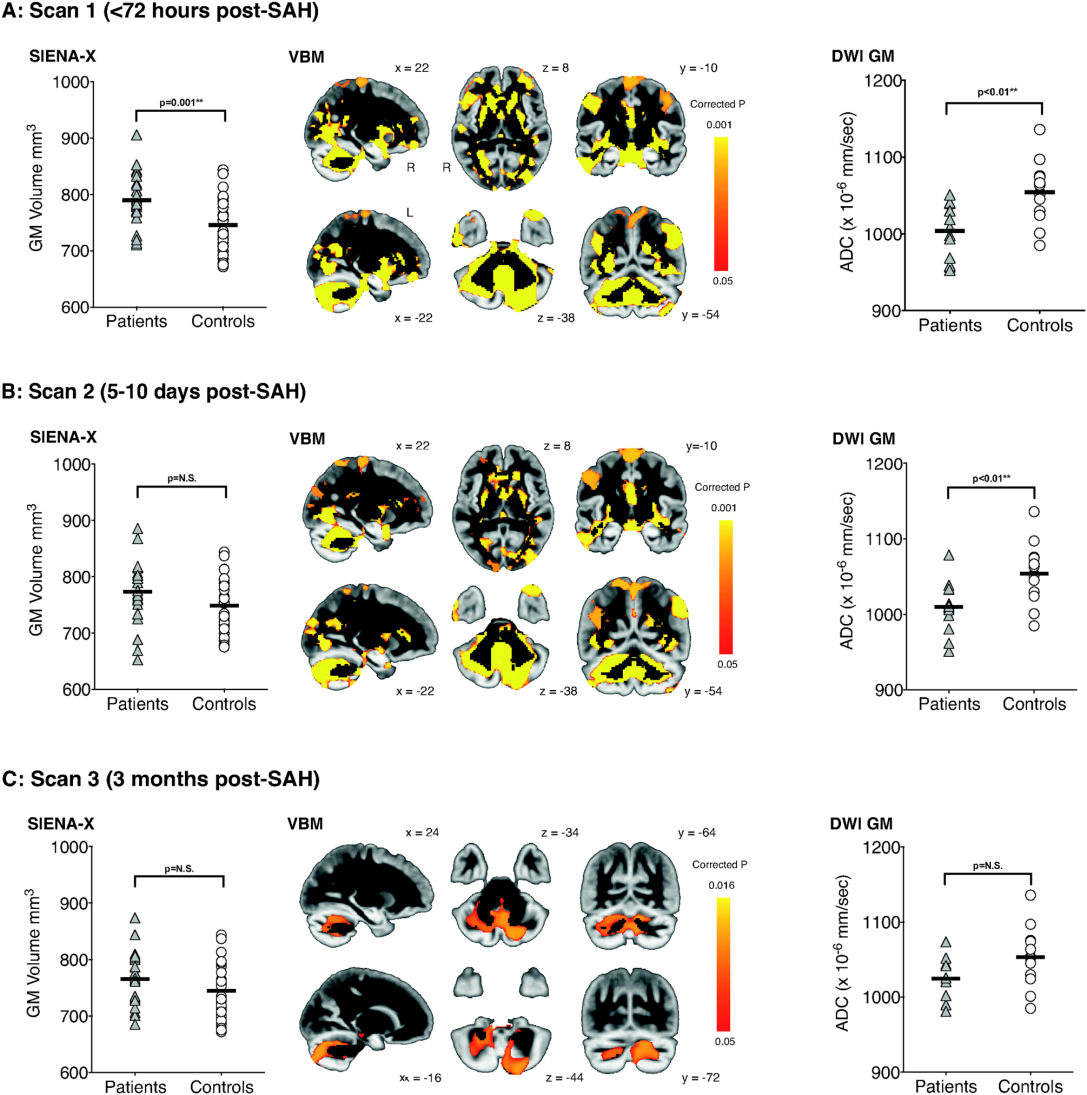
Higher GM volume (global: SIENA-X, local: VBM) and lower GM DWI between SAH patients and healthy controls in acute phase subside at 3 months post-SAH, except in the cerebellum. Results are given for each timepoint (**A** acute phase, < 72 h post-SAH; **B** 5–10 days post-SAH; and **C** 3 months post-SAH). Left, plots showing results from the SIENA-X analyses looking at mean whole-brain GM volume. Middle, results from the voxel-by-voxel VBM analysis showing in red-yellow the GM areas where patients have apparent higher volume (p < 0.05-TFCE-corrected for multiple comparisons, overlaid onto the average of all 54 GM volume images). Right, plots showing the mean ADC values in a whole-brain GM mask. L is R.

SIENA-X analysis showed significant apparent *higher* total brain volume, whole-brain GM and WM volumes in patients compared with healthy controls, while ventricular CSF volumes showed a trend towards higher values in patients (Table 2, Fig. 2A). In line with the apparent higher whole-brain GM volume, we found a significantly lower mean GM ADC values for the patients compared with controls (Table 2, Fig. 2A).

**Table.2:** Global MRI brain measures at each assessment (volumetric: SIENA-X and water diffusion: diffusion-weighted imaging—DWI).

	Patients (mean ± std)	Controls (mean ± std)	p
**Assessment 1 (< 72 h post-SAH)**			
SIENA-X			
Total brain volume (mm^3^)	1521 ± 66	1461 ± 90	**0.01**
Grey matter volume (mm^3^)	790 ± 44	746 ± 50	**< 0.01**
White matter volume (mm^3^)	731 ± 28	706 ± 54	**0.03**
Ventricular CSF volume (mm^3^)	45 ± 20	37 ± 8	0.06
DWI			
Mean grey matter ADC (× 10^−6^ mm^2^/s)	1004 ± 35	1054 ± 40	**< 0.01**
**Assessment 2 (5–10 days post-SAH)**			
SIENA-X			
Total brain volume (mm^3^)	1502 ± 68	1455 ± 99	0.07
Grey matter volume (mm^3^)	773 ± 54	748 ± 48	0.05
White matter volume (mm^3^)	729 ± 29	707 ± 54	0.09
Ventricular CSF volume (mm^3^)	46 ± 20	37 ± 8	**0.03**
DWI			
Mean grey matter ADC (× 10^−6^ mm^2^/s)	1010 ± 33	1054 ± 40	**0.001**
**Assessment 3 (3 months post-SAH)**			
SIENA-X			
Total brain volume (mm^3^)	1480 ± 61	1461 ± 90	0.39
Grey matter volume (mm^3^)	765 ± 45	748 ± 50	0.21
White matter volume (mm^3^)	718 ± 23	708 ± 55	0.43
Ventricular CSF volume (mm^3^)	55 ± 26	37 ± 8	**0.009**
DWI			
Mean grey matter ADC (× 10^−6^ mm^2^/s)	1025 ± 26	1053 ± 41	0.06

Significant values are in bold.

Voxel-by-voxel regional analysis using VBM showed that this apparent higher GM volume in the patients was widespread, particularly in regions including the anterior cingulate/paracingulate gyrus, primary motor cortex, left supramarginal and angular gyrus, fusiform gyrus (especially on the right), basal ganglia (caudate and putamen bilaterally) and cerebellum (Fig. 2A).

##### Assessment 2: 5–10 days post-SAH

There were trends towards higher total brain volume, whole-brain GM or WM volume in patients compared with healthy controls, while ventricular CSF volume was this time significantly higher in the patients (Table 2, Fig. 2B). There was a reduction in mean GM ADC in the patients compared with healthy controls (Table 2, Fig. 2B).

VBM analysis revealed apparent higher GM volume that was less marked than at Assessment 1 but still widespread, and especially prominent in the angular gyrus, fusiform gyrus and most markedly in the cerebellum (Fig. 2B).

##### Assessment 3: Three months post-SAH

At 3 months following SAH, there was no significant difference in total brain volume, whole-brain GM or WM volume between patients and controls. However, there remained a significant increase in ventricular CSF volume in patients when compared with controls (Table 2, Fig. 2C). There was also no longer any significant difference in whole-brain GM ADC (Table 2, Fig. 2C).

However, the regional VBM analysis highlighted a persistent and significantly higher GM volume in patients compared with controls in the cerebellum (Fig. 2C). These remaining GM abnormalities were, on closer inspection, mainly localised bilaterally in the cognitive cerebellar lobule VII: mainly in Crus II, but also Crus I and VIIb to a lesser extent.

#### Longitudinal analyses within the patient group

These can be found in the Supplementary Material.

#### Cognitive outcome analyses

**Figure 3:**
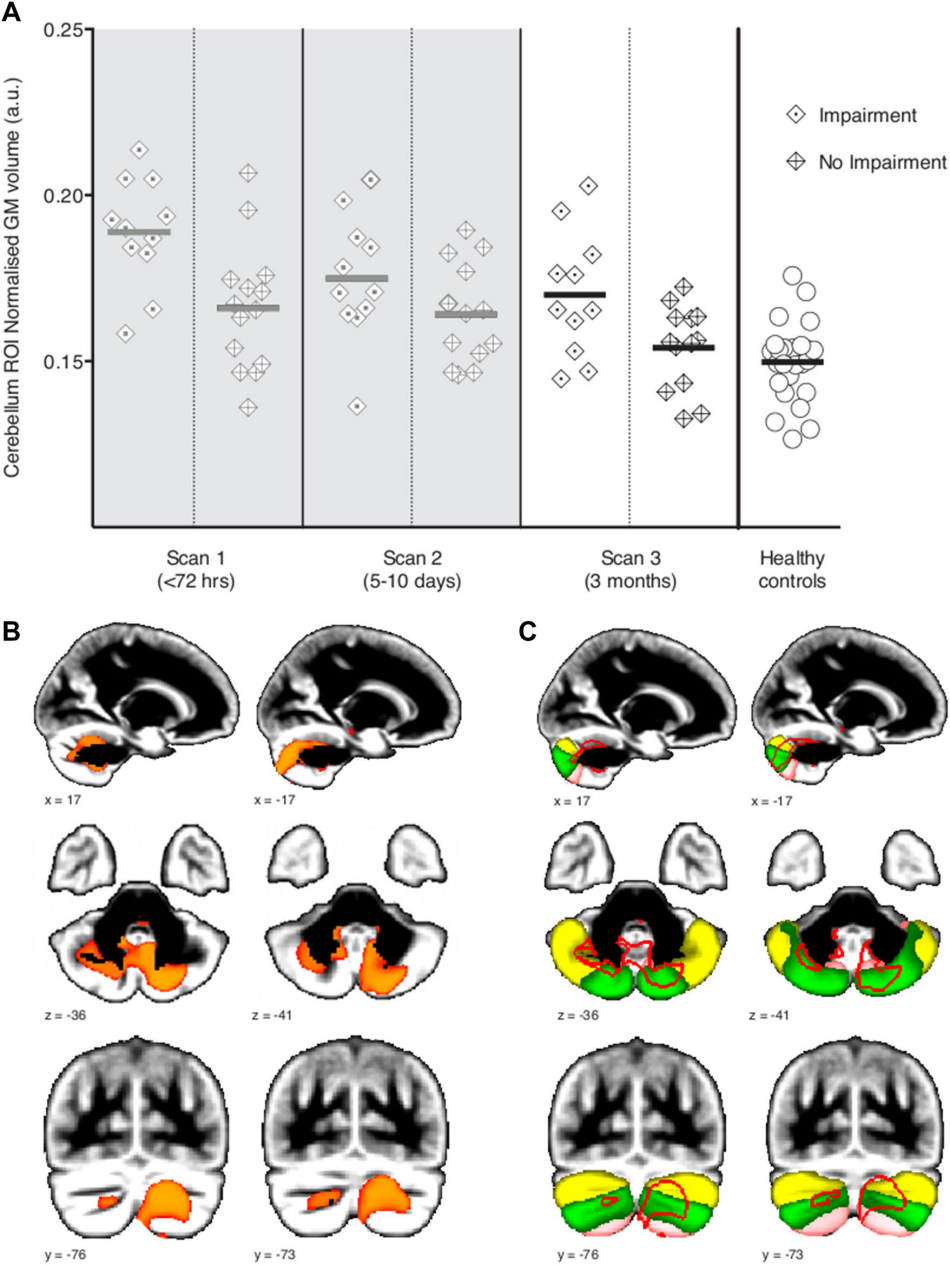
Residual GM volume differences in the posterior cerebellum at 3 months post-SAH are related to patients with cognitive impairment. (**A**) GM volume values in the significant cerebellum residual abnormalities. While the values are on average similar between those patients without cognitive impairment and the healthy controls, they are clearly higher in those with cognitive impairment (calculations done using the weighted average in the supra-threshold cluster shown in **B**). For visualization only, we also present those same values for the first two timepoints (< 72 h post-SAH and 5–10 days post-SAH; in greyed areas). (**B**) Results from the regional GM analysis (VBM) at 3 months post-SAH. Patients have higher GM volumes in the posterior, cognitive cerebellum (red-yellow, p < 0.016 TFCE-corrected) encompassing regions of crus I, crus II and VIIb bilaterally (as shown in **C**, in yellow, green and pink, respectively from a probabilistic atlas). L is R.

We sought to test the hypothesis that the remaining differences found in the patients at 3 months post-SAH (scan 3) (Fig. 3B) might be imputable to the patients with worse cognitive outcomes, as these differences were observed specifically in cognitive regions of the cerebellum, mainly Crus II, Crus I and VIIb^16^ (Fig. 3C).

In the cerebellar region of interest (ROI) defined by this significant, apparent increase in GM volume in the *entire* patient group compared with the healthy controls (“scan3-ROI”), those patients with cognitive impairment had higher GM volumes indeed compared to those without, who seemed to have GM volumes similar to those of healthy controls (Fig. 3A). Cohen’s d calculated between patients with and without impairment for the GM volume of the cerebellum scan 3-ROI was 0.72—suggesting a medium to large effect size difference between the two groups^17^.

**Figure 4:**
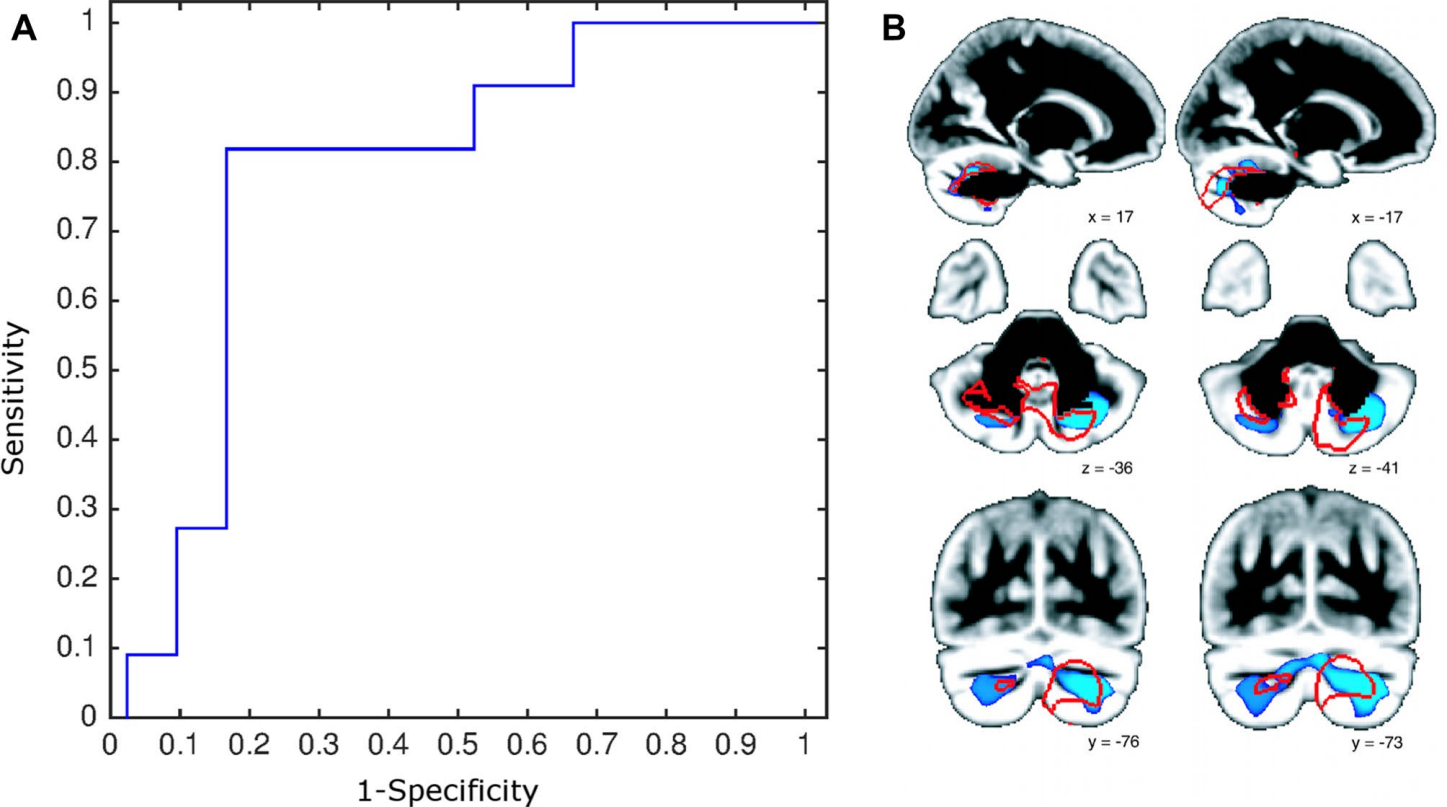
Posterior cerebellum volume in the acute phase post-SAH predicts future neurocognitive outcomes. (**A**) Receiver operator characteristic (ROC) curve for neurocognitive impairment (weighted average GM volume values from the acute-phase scans < 72 h using the supra-threshold cluster shown in Fig. 3B as a region of interest). Maximum accuracy of 84% is achieved using leave-one-out cross-validation with a threshold of 0.1792, and at this point on the ROC curve, the sensitivity is 82% and the specificity is 86%. (**B**) Direct comparison at < 72 h post-SAH reveal apparent higher cerebellar volume in patients with cognitive impairment compared to those without (blue, p < 0.001 TFCE-corrected). These regions broadly correspond to those showing residual abnormalities at 3 months.

Next, we investigated whether these cognitive outcomes at 3 months post-SAH could have actually been *predicted* from looking, in the acute phase, at this same region of the brain in the patients *only*. By extracting the normalised GM volume values at scan 1 using this cerebellum scan 3-ROI and using leave-one-out cross-validation, we found that the GM cerebellar values yielded a maximum accuracy of 84% to discriminate patient with vs. without cognitive impairment, which could be achieved with a threshold of 0.1792. At this point on the ROC curve, the sensitivity was 82% and the specificity is 86% (and a balanced accuracy of 84%). The area under the curve (AUC) for the ROC was 0.805, with 95% confidence interval from 0.576 to 0.921 (Fig. 4A).

A *direct* VBM analysis at scan 1 comparing patients with and without future cognitive impairment confirmed that those who went on to develop impairment at 3 months post-SAH already showed a significant apparent higher GM volume in a cerebellar region which overlapped with the cerebellar ROI defined above, but which also extended more prominently into lobules VIIb and VIIIa (Fig. 4B).

#### Exploratory UK Biobank analyses

Three genetic findings were related to the GM volume measured in the cerebellum of the ~ 10,000 UK Biobank participants: *SLC39A8*/*ZIP8* which has been linked to higher risk of cardiovascular death^18,19^, *SLC44A5* an important gene for metabolism of lipids and lipoproteins^20^, and *PAPPA*, which critically codes for a protein that acts in particular as a predictor of poor outcome in transient ischemic attack and ischemic stroke^21^.

When specifically investigating the volume of cerebellar regions strongly linked to cognitive impairment in the SAH patients (lobule VII: Crus I, Crus II and lobule VIIb), we once again uncovered significant associations in the UK Biobank participants with *PAPPA* (Crus I), but also with *EXOC5* and *TSHZ1* (Crus II). In turn, the most strongly associated locus in an intron of *PAPPA* (rs72754248, p = 1.4 × 10^−17^) correlated significantly with two primary causes of death (ICD10 J43.9: Emphysema, p = 2 × 10^−5^; ICD10 X59.5: Accidental exposure to other and unspecified factors, p = 3.6 × 10^−6^). The strongest locus in proximity to *EXOC5* (rs76684157, p = 9.1 × 10^−10^) was associated at a trend level with the medication nicorandil, a vasodilatory drug and anti-anginal agent, being taken by the UK Biobank participants (p = 1.8 × 10^−4^), while the locus close to *TSHZ1* (rs4891262, p = 7.4 × 10^−10^) was associated at a trend level with the medication spironolactone, given to treat high blood pressure and heart failure (p = 2 × 10^−4^).

Furthermore, we uncovered from our latest, extended genetic-imaging UK Biobank study^22^, further associations of lobule VII with many additional variants principally related to cardiovascular disease and risk factors, including rs56300220, a locus in an exon of *LRP8*, rs2199936, in an exon of *ABCG2*, as well as two introns in *MFHAS1*.

## Discussion

This study is the first to use MRI to quantify the acute pathophysiological consequences of SAH in specifically good grade patients. We demonstrate that MRI brain scanning in the acute stage accurately predicts the *future* cognitive impairment in patients who, crucially, appeared clinically indistinguishable on presentation from those making full recovery.

### Good grade SAH leads to global brain changes quantifiable with acute MRI

The incidence of global cerebral oedema specifically in good grade SAH patients (WFNS I and II) is unknown. Results of recent studies suggest that the incidence may be in the region of 57–81% of all clinical grade of SAH patient^13,23^. However, it remains currently difficult to objectively quantify oedema with routine clinical neuroimaging such as CT^9,24^. We identified a significant apparent increase in GM volume within the first 72 h post-SAH compared with gender- and age-paired healthy controls (Fig. 2A). Median values for the patients were 80% higher in GM volume and 100% lower in ADC value compared with those seen in the control group. Both these proxy in vivo measures are highly indicative of cytotoxic cerebral oedema, with cellular swelling causing an increase in volume and decrease in water diffusion. Global cerebral oedema after SAH is known to be associated with worse initial clinical condition^23^ and early brain metabolic distress^25^.

### Persistent abnormalities in cerebellar volume after 3 months relate to cognitive impairment

Despite having the most potential for neurological recovery, few studies have specifically focused on cognitive outcomes in patients with good clinical grade SAH (i.e. WFNS 1 and 2)^26,27^. As a result, subtle cognitive deficits that exert a disproportionately significant impact on quality of life can go undiagnosed. Our results highlight that the incidence of cognitive impairment at 3 months post-SAH remains high in patients with good grade SAH. Almost 50% of patients were defined as being cognitively impaired with significant deficits in the domains of executive functioning, processing speed and memory (Fig. S1). This is in agreement with other studies that have suggested that the incidence of these deficits ranges from 7 to 62% of patients^28^.

It is highly likely that damage to the cerebellum influence the degree of cognitive impairment exhibited by patients at 3 months post-SAH. Our voxel-by-voxel analysis highlighted a persisting higher GM volume mainly localized to an ROI of lobule VII (Crus I, II and lobule VIIb) bilaterally (Fig. 2C). This difference was driven by those patients who had developed cognitive impairment post-SAH (Fig. 3A). There is increasing convergent and multi-modal evidence from both basic science and clinical studies that the cerebellum, and especially lobule VII, has a key role in higher cognitive functions^16,29,30^. In particular, functional imaging studies have highlighted the role of Crus I in executive functions^31^, while lesions to Crus II result in impaired verbal fluency^32^.

One plausible hypothesis for the increased GM volume is that this represents residual cerebral oedema. The exact time course of oedema resolution after SAH remains unknown. Studies in animals suggest that cerebral oedema following traumatic brain injury resorbs over a period of weeks^33^. An alternative explanation may be that the study population of good grade SAH patients included a large number with high blood load (modified Fisher grade 4 and 5). The recumbent nature of many patients during the acute admission post-SAH might have also led the cerebellum, especially its most posterior part (precisely lobule VII), to sustain increased injury due to a gravity effect and subsequent haemotoxicity of this higher blood load.

### Genetic findings from UK Biobank related to the cerebellum

It is worth noting that, using the same voxel-by-voxel measure in ~ 40,000 UK Biobank participants, the cerebellar GM volume, and especially that of lobule VII, is repeatedly associated with multiple genes involved in cardiovascular events. In particular, a genetic variant in *ZIP8*, which is associated with the GM volume of most of Crus II (described in Elliott et al.^19^ Extended Data Fig. 1), has been linked to higher risk of cardiovascular death^18^. Similarly, *PAPPA*, which is in particular related to Crus I, Crus II and lobule VIIb, has been shown increasingly to have a consistent role in cardiovascular disease^34,35^. Serum PAPP‐A concentration notably emerges as a predictor of risk for poor outcome in transient ischemic attack and ischemic stroke^21^. We further uncovered associations between the regions of lobule VII and exonic variants of *LRP8* and *ABCG2*, both genes being implicated in familial and premature coronary artery disease and myocardial infarction, and cardiovascular disease risk factors, respectively^36,37^. Finally, two intronic variants of *MFHAS1* that we found associated with lobule VII have been shown, in previous GWAS, to be significantly involved in the use of medication acting on the renin-angiotensin system, as well as with Type II diabetes^38,39^. Persisting higher cerebellar volume thus appears to be a meaningful marker of the deleterious influence of proteins relevant to cardiovascular disease, and a potential factor in the cognitive impairment subsequently developed by those who had higher volumes in the acute phase. The question remains whether such specific vulnerability of the posterior cerebellum predates the SAH event.

### Cerebellum GM volume during acute phase predicts cognitive outcomes 3 months post-SAH

There was no significant difference between patients with and without future cognitive impairment in age, gender, hydrocephalus and angiographic vasospasm, clinical features such as Fisher score, or, importantly, in the location of the aneurysm (Table 1). Interestingly, the only demographic or clinical difference was that patients with future impairment actually demonstrated *lower* WFNS scores (i.e. *better* clinical condition upon admission, Table 1). However, when using an ROI of those remaining cerebellar abnormalities seen at scan 3 across all patients, it was actually possible to predict within the first 72 h post-rupture which patients went on to poor cognitive outcomes with 84% accuracy and 86% specificity (Fig. 4A). A *direct* comparison between patients with and without future impairment based on their brain scans in the acute phase confirmed higher GM volume in the cerebellum (mainly lobule VII and VIIIa) in the patients with future poorer outcome (Fig. 4B). This demonstrates the promise of measuring brain tissue alterations in the posterior cerebellum to stratify patients very early in their clinical pathway.

In summary, in an exploratory sample of good grade SAH patients compared to healthy controls, we identified a region of the posterior cerebellum for which acute changes on MRI were associated with cognitive impairment. The high sensitivity of our voxel-by-voxel approach to both global and regional, localised changes in GM volume also demonstrates that significant cerebral oedema occurs acutely even in good grade SAH as a result of early brain injury. Acute damage to the posterior cerebellum is potentially a pathophysiological mechanism that contributes towards subsequent cognitive impairment and requires further prospective study. Whilst further investigation will be required to confirm causality, use of this finding as a risk stratification biomarker in SAH is promising.

## Methods

The study was designed as a longitudinal, prospective cohort study. Approval for the study was granted by the local National Research Ethics Service committee (NRES Committee South Central—Berkshire: 11/SC/0519). All methods were performed in accordance with the relevant guidelines and regulations and in accordance with the Declaration of Helsinki. Patients, and age/gender-paired healthy controls, were recruited from a tertiary neurosurgical centre. Informed written consent was obtained from all participants or a nominated consultee.

Patients were eligible for recruitment to the study according to the following inclusion criteria:

1. male or female, aged between 18–80,
2. WFNS Grade I and II SAH and.
3. presentation to emergency medical services and successful aneurysm occlusion within 72 h of index headache.

Patients were assessed on three occasions post-SAH: Assessment 1 was at an acute stage within 72 h post-rupture, Assessment 2 was between 5–10 days post-rupture and Assessment 3 was performed 3 months post-rupture. Healthy controls attended a session during which MRI scanning, cognitive and clinical measures were obtained.

Imaging data, acquired using a 3 T Verio MR, included:

1. Whole-brain structural T1-weighted sequence to measure grey matter (GM) volume:
  Globally: whole-brain GM, white matter (WM) and ventricular cerebrospinal fluid volumes were estimated in each scan using the FSL tool SIENAX^40^.
  Regionally: an optimized voxel-based morphometry (VBM) analysis was then undertaken to identify regional, localised differences in GM volume using FSL-VBM^14^.
2. Diffusion-weighted imaging (DWI) to measure magnitude of water diffusion: using the same VBM standard space, mean GM ADC values were extracted for each scan.

All voxel-wise statistical analyses were carried out using permutation testing in FSL^41^.

Following discharge, patients were invited to return for a follow-up appointment at 3 months post-SAH. This involved a comprehensive set of cognitive assessments carried out by trained assistant psychologists, under the supervision of a consultant clinical neuropsychologist.

Individuals’ cognitive test scores that fell at or below the 5th percentile (equivalent to a z-score ≤ 1.65) were identified and classified as impaired scores (or “deficits”)^42^. Neurocognitive impairment was then defined as presence of two or more impaired test scores of the five cognitive domains (see Supplementary Material)^42^. The complete assessment battery is outlined in Table S1.

MRI data were analysed according to four main analyses:

1. Cross-sectional group comparison: we compared patients and controls at each time-point of the three assessments.
2. Longitudinal comparison within the patient group: we compared scans at Assessment 1 with scans at Assessments 2 and 3.
3. Cognitive outcome analysis: we assessed whether the poor cognitive outcome observed a posteriori in certain patients at 3 month (cognitive impairment as defined above) could be predicted from their scans in the acute phase.
4. UK Biobank analysis: we related our brain imaging results from 3. with those imaging findings from the UK Biobank (n = ~ 10,000), to investigate the possible genetic mechanisms underpinning the anatomical differences observed in those with poor cognitive outcome.

Full details of the “Materials and methods”, including Statistical Analyses, are available in the Supplementary Material.

## Supplementary Information

Supplementary Information.

## Data Availability

http://big.stats.ox.ac.uk/. http://www.nealelab.is/blog/2017/7/19/rapid-gwas-of-thousands-of-phenotypes-for-337000-samples-in-the-uk-biobank.
