# Supplementary material for: Early brain injury and cognitive impairment after aneurysmal subarachnoid haemorrhage
Source: Sci Rep. 2021 Dec 1;11:23245. doi: 10.1038/s41598-021-02539-x (PMC8636506; doi:10.1038/s41598-021-02539-x)
Supplement: Supplementary file 1 — Supplementary Information. [file 41598_2021_2539_MOESM1_ESM.docx]

**ONLINE SUPPLEMENTARY MATERIAL**

**Early brain injury and cognitive impairment after aneurysmal subarachnoid haemorrhage**

MJ Rowland DPhil^1.2,3^, P Garry FRCA^1,2^, M Ezra FRCA^1,2,3^, R Corkill FRCR^2^, I Baker^2^, P Jezzard PhD^3^, J Westbrook FRCA^,2^, G Douaud PhD^3^*, KTS Pattinson DPhil^1,2,3^*

^1^Nuffield Division of Anaesthetics, Nuffield Department of Clinical Neurosciences, University of Oxford, John Radcliffe Hospital, Oxford OX3 9DU, United Kingdom

^2^Neurosciences Intensive Care Unit, Oxford University Hospitals NHS Trust, Oxford OX3 9DU, United Kingdom

^3^Wellcome Centre for Integrative Neuroimaging, Nuffield Department of Clinical Neurosciences, University of Oxford, UK

* denotes each author contributed equally to this work

**DEFINITIONS**

Aneurysm rupture was defined as primary headache associated with presentation to emergency medical services.

**EXTENDED MRI METHODS**

**MRI sequences:**

1. To measure grey matter (GM) volume, a whole-brain structural T1-weighted MPRAGE was acquired with 1.5 × 1.5 × 1.5 mm resolution; TR 1780 ms; TI 900 ms; TE 4.4 ms; scan time 2 mins 22 s.
2. To measure magnitude of water diffusion, diffusion-weighted imaging (DWI) echo planar, spin echo sequence was acquired with voxel size 1.8 × 1.8 × 5 mm; TR 5300 ms; TE 91 ms; b values 0 and 1000 s/mm^2^, scan time 1 min. Corresponding b0 fieldmap images were acquired at each scan point to aid scan registration by correcting for echo-planar imaging distortions.

**Measuring whole-brain and regional GM volume**

**Whole-brain**: Whole-brain grey matter (GM) white matter (WM) and ventricular cerebrospinal fluid volumes were estimated in each scan using the FSL tool SIENAX.^1^ SIENAX starts by extracting brain and skull images from the single whole-head input data.^2^ The brain image is then affine-registered to MNI152 space^3,4^ (using the skull image to determine the registration scaling); this is primarily done to obtain the volumetric scaling factor, which can then be used for normalisation of head size. Next, tissue-type segmentation with partial volume estimation is carried out^5^ in order to calculate total volume of brain tissue, including separate estimates of volumes of GM, WM and ventricular cerebrospinal fluid (CSF).

**Regional**: An optimized voxel-based morphometry (VBM) analysis was then undertaken to identify regional, localised differences in whole brain GM volume (FSL-VBM: <http://fsl.fmrib.ox.ac.uk/fsl/fslwiki/FSLVBM>^6^). All images were processed following the same pipeline: first, structural images were brain-extracted and GM-segmented before being registered to the MNI152 standard space using non-linear registration. The resulting images were averaged and flipped along the x-axis to create a left-right symmetric, study-specific GM template. Second, all native GM images were non-linearly registered to this study-specific template and "modulated" to correct for local expansion (or contraction) due to the non-linear component of the spatial transformation. The modulated GM images were then smoothed with an isotropic Gaussian kernel with a sigma of 3mm.

**Measuring magnitude of water diffusion**

Apparent diffusion coefficient (ADC) maps obtained from the scanner were brain extracted.^7^ Registration of the b0 image to the native T1-weighted image was estimated for each subject with a multi-step linear registration masking out ventricular CSF intensity and using boundary-based registration.^3,8,9^ Next, we combined this within-subject registration with the non-linear registration of each subject’s GM segmented image to the VBM template. At the end of this registration process, ADC maps were therefore in the same space as the one generated using VBM to investigate regional GM volume. A GM mask from the VBM analysis (thresholded at 0.5 and binarised) was then used to extract data for mean GM ADC from each scan.

**Physiological data collection during MRI scanning**

Physiological data were also collected during MRI scanning sessions. Expired oxygen (O_2_) and carbon dioxide (CO_2_) were collected to calculate PETO2/PETCO2 using disposable nasal cannulae and an AD Instruments (Sydney, Australia) gas analyser sampling at 50Hz. Additionally, pulse oximetry and heart rate (MR Systems, USA) and non-invasive blood pressure were recorded every 5 mins during the scan session (Invivo Research, Florida, USA).

**COGNITIVE OUTCOMES**

**Assessment of cognitive outcomes**

**At Assessment 1 and 2:** While inpatients, recruited subjects underwent daily clinical assessment of neurological status.

**At Assessment 3:** Following discharge, patients were invited to return for a follow up appointment at 3 months post-SAH. This involved a set of cognitive assessments carried out by trained assistant psychologists, under the supervision of a consultant clinical neuropsychologist.

Firstly, a rapid neurocognitive screen using the Addenbrookes Cognitive Examination (ACE-III) was conducted. This assessed five cognitive domains: attention/orientation, memory, verbal fluency, language and visuospatial abilities.^10^

Secondly, we used an assessment battery from a sub-study of The International Subarachnoid Aneurysm Trial (N-ISAT) that was designed to assess neurocognitive function after SAH.^11^ However, to keep the time required to complete the battery to a reasonable level and improve patient compliance and decrease the likelihood of missing datasets, test measures were selected from the overall battery which correlated most highly with five of these domains, as per Scott et al.^12^ These were: verbal memory, general verbal skills, processing speed, nonverbal skills/memory, and executive skills.

Finally, we included the Matrix Reasoning (subtest of the Weschler Abbreviated Scale of Intelligence - WASI) to provide overall IQ scores, Stroop and Trail Making Tests to further assess processing speed and executive function and premorbid IQ was estimated using the re-standardised National Adult Reading Test (NART; 2nd Edition).^13^

Control subjects underwent the same ACE-III testing as that received by the patients at each Assessment, on the same day as they had their MRI scan.

The raw data set from each test was then transformed into standardized z-scores (with a mean of 0 and a standard deviation of 1.0) relative to age-matched normal populations, employing standard reference manuals and materials for each test. The only exception to this was the CANTAB Intradimensional/Extradimensional Shift (ID/ED) task, which taps the executive component of set-shifting or cognitive flexibility, and was classed as “pass” or “fail”.

In line with widely accepted clinical practice and as used in the neuropsychological outcomes substudy of the International Subarachnoid Aneurysm Study (N-ISAT)^11^, individuals’ test scores that fell at or below the 5th percentile (equivalent to a z-score ≤ 1.65) were then identified and classified as impaired scores (or “deficits”). “Fail” of the CANTAB ID/ED task was classified as an impaired score. Neurocognitive impairment was then defined as presence of two or more impaired test scores of the five cognitive domains described above.^11^ The complete assessment battery is outlined in **Table S1**.

**EXTENDED STATISTICAL ANALYSIS METHODS**

All voxel-wise statistical analyses were carried out using permutation testing in FSL.^14^ Additional statistical analyses were conducted using Prism 6 (GraphPad Software) and R [(http://www.r-project.org/).](http://www.r-project.org/))

**MRI data analysis**

1. **Cross-sectional group comparison analyses**

SIENAX and DWI group comparisons between patients and controls were carried out using unpaired Students t-test for parametrically distributed data, or Mann-Whitney U test for non-parametrically distributed data (normality of the data was tested using the Shapiro-Wilk test).

Voxelwise statistical differences between patients and controls for the GM regional volumetric analysis (VBM) at each scan point were assessed with permutation testing in the FSL randomise tool^14^ using 5,000 permutations and Threshold-Free Cluster Enhancement (TFCE)^15^, to fully correct for multiple comparisons across space. Results were considered significant at p<0.05 TFCE-corrected.

1. **Longitudinal analyses within the patient group**

Longitudinal comparisons within the patient group for the SIENAX and DWI data were analysed using a repeated measures ANOVA with statistical significance set at p<0.05. For the longitudinal voxelwise GM volumetric analyses within the patient group, difference maps were calculated between scan 1 and scan 2 or 3 to account for repeated measure. These difference maps (one per patient) were then entered into a one sample t-test, and results considered significant at p<0.05 TFCE-corrected.

1. **Cognitive outcome analyses**

Further statistical analysis on MRI data was performed by dividing patients by outcome into cognitively impaired and cognitively non-impaired groups (see criteria above), to assess the potential of MRI biomarkers in predicting cognitive impairment.

Weighted average GM volume values were obtained from the T1-weighted scans from Assessment 1 (<72 hours post-SAH) in all 25 patients whose cognitive outcome was known (those completed the cognitive battery of tests 3 months post-SAH), using a region of interest (ROI) in the cerebellum. This ROI was defined as the only region showing significant difference when comparing the group of patients at 3 months post-SAH with the healthy controls (in effect achieving out-of-sample testing).

We then used a leave-one-out cross-validation and receiver-operating characteristic (ROC) analysis to estimate the maximum accuracy with which patients who would become cognitively impaired and non-impaired (Assessment 3) could be distinguished based on these GM values at the acute-stage (Assessment 1).

1. **UK Biobank genetic analyses**

We first considered whether any of the findings from our previous study correlating 3,144 brain imaging features with genetic variants in ~10,000 UK Biobank participants^16^ related to the same brain regions identified in our previous analysis of poor cognitive outcome.

Second, we looked for the genetic correlates of the main regions specifically showing differences between those with and without cognitive impairment, using our largest UK Biobank cohort to date.^17^ Results were corrected for multiple comparisons at GWAS levels for the genetic loci (p<5×10^−8^). For association of these genetic variants with selected clinical correlates, we corrected for multiple comparisons across these phenotypes (n=1,942 in total; Treatment/medication: 778, Non-cancer illness: 341, Primary cause of death: 141, Family illnesses: 40, ICD10 codes: 642). For more details, please see URLs at the end of the manuscript.

**EXTENDED RESULTS**

**Longitudinal analyses within the patient group**

**Table S3** highlights the analysis of the longitudinal changes in SIENAX and DWI analysis in patients between Assessment 1, 2 and 3. Between Assessment 1 and Assessment 2, there was no significant difference in GM, WM or ventricular CSF volumes using SIENAX. Between Assessment 1 and Assessment 3, SIENAX analysis showed a significant reduction in total brain volume, GM volume and WM volume. Associated with these decreased brain volumes was a significant increase in vCSF volume between Assessment 1 and Assessment 3. Between Assessment 1 and 2, there was no significant change in whole-brain GM ADC while an increase in whole-brain GM ADC values was seen in patients between Assessment 1 and Assessment 3.

The regional VBM analysis demonstrated no significant difference between Assessment 1 and 2. There was a widespread apparent decrease in GM volume between Assessments 1 and 3 (Figure S3).

**EXTENDED DISCUSSION**

Although the large variability in the prevalence of cognitive deficits can partly be attributed to the heterogeneous nature of SAH e.g., aneurysm location, blood load and incidence of hydrocephalus/DCI etc., some of this variability may also be due to the absence of standardized criteria for diagnosing patients with cognitive impairment. To address this, we specifically used the same criteria used in the two largest prospective trials assessing cognitive impairment post-SAH.^11,18^ One limitation (in terms of sensitivity, but not statistical validity) of our study is the relatively small number of participants. Imaging data were however analysed using permutation-based non-parametric inference fully corrected for multiple comparisons over space (number of voxels, for the whole-brain voxel-by-voxel analyses). Investigation of other data was also conducted using non-parametric techniques when data were non-normally distributed. As a consequence of the small sample^19^, the actual effect sizes observed in our study were comparatively larger than it would have needed to be for a larger group, with for instance 80 to 100% difference between patients and healthy controls in their imaging measures during the acute phase.

The origin of the signal differences observed in the brain tissue cannot be confirmed with our non-invasive imaging methods. These could be affected by residual blood in the subarachnoid space and ventricles post-SAH; however, the majority of the voxel-by-voxel differences were not observed in the vicinity of the ventricles (e.g., basal ganglia, cerebellum and precuneus).

**REFERENCES**

1. Smith, S. M. *et al.* Accurate, robust, and automated longitudinal and cross-sectional brain change analysis. *NeuroImage* 17, 479–489 (2002).

2. Smith, S. M. Fast robust automated brain extraction. *Human Brain Mapping* 17, 143–155 (2002).

3. Jenkinson, M. & Smith, S. A global optimisation method for robust affine registration of brain images. *Medical image analysis* 5, 143–156 (2001).

4. Jenkinson, M., Bannister, P., Brady, M. & Smith, S. Improved optimization for the robust and accurate linear registration and motion correction of brain images. *NeuroImage* 17, 825–841 (2002).

5. Zhang, Y., Brady, M. & Smith, S. Segmentation of brain MR images through a hidden Markov random field model and the expectation-maximization algorithm. *IEEE transactions on medical imaging* 20, 45–57 (2001).

6. Douaud, G. *et al.* Anatomically related grey and white matter abnormalities in adolescent-onset schizophrenia. *Brain : a journal of neurology* 130, 2375–2386 (2007).

7. Smith, S. M. Fast robust automated brain extraction. *Human Brain Mapping* 17, 143–155 (2002).

8. Jenkinson, M., Bannister, P., Brady, M. & Smith, S. Improved optimization for the robust and accurate linear registration and motion correction of brain images. *NeuroImage* 17, 825–841 (2002).

9. Greve, D. N. & Fischl, B. Accurate and robust brain image alignment using boundary-based registration. *NeuroImage* 48, 63–72 (2009).

10. Mioshi, E., Dawson, K., Mitchell, J., Arnold, R. & Hodges, J. R. The Addenbrooke’s Cognitive Examination Revised (ACE-R): a brief cognitive test battery for dementia screening. *International Journal of Geriatric Psychiatry* 21, 1078–1085 (2006).

11. Scott, R. B. *et al.* Improved Cognitive Outcomes With Endovascular Coiling of Ruptured Intracranial Aneurysms: Neuropsychological Outcomes From the International Subarachnoid Aneurysm Trial (ISAT). *Stroke* 41, 1743–1747 (2010).

12. Scott, R. B., Eccles, F., Lloyd, A. & Carpenter, K. From multidimensional neuropsychological outcomes to a cognitive complication rate: The International Subarachnoid Aneurysm Trial. *Trials* 9, 13 (2008).

13. Willshire, D., Kinsella, G. & Prior, M. Estimating WAIS-R IQ from the national adult reading test: A cross-validation. *Journal of Clinical and Experimental Neuropsychology* 13, 204–216 (1991).

14. Winkler, A. M., Ridgway, G. R., Webster, M. A., Smith, S. M. & Nichols, T. E. Permutation inference for the general linear model. *NeuroImage* 92, 381–397 (2014).

15. Smith, S. M. & Nichols, T. E. Threshold-free cluster enhancement: addressing problems of smoothing, threshold dependence and localisation in cluster inference. *Neuroimage* 44, 83–98 (2009).

16. Elliott, L. T. *et al.* Genome-wide association studies of brain imaging phenotypes in UK Biobank. *Nature* 562, 210–216 (2018).

17. Smith, S. M. *et al.* An expanded set of genome-wide association studies of brain imaging phenotypes in UK Biobank. *Nat Neurosci* 24, 737–745 (2021).

18. Wong, G. K. C. *et al.* Cognitive domain deficits in patients with aneurysmal subarachnoid haemorrhage at 1 year. *Journal of Neurology, Neurosurgery & Psychiatry* (2013) doi:10.1136/jnnp-2012-304517.

19. Friston, K. Ten ironic rules for non-statistical reviewers. *NeuroImage* 61, 1300–1310 (2012).

**Table S1: Complete neurocognitive testing battery at Assessment 3 (3 months post-SAH)**

| **NEUROCOGNITIVE DOMAIN** | **TEST** |
| --- | --- |
| **Premorbid IQ:** | National Adult Reading Test (NART 2^nd^ Edition) |
| **Rapid screen of neurocognitive performance** | Addenbrookes Cognitive Examination Revised |
| **Verbal Memory:** | Adult Memory and Information Processing Battery (AMIPB - mean of immediate and delayed recall)  California Verbal Learning Test (CVLT) |
| **General Verbal Skills:** | Weschler Abbreviated Scale of Intelligence (WASI):   - Vocabulary - Similarities - Matrix Reasoning |
| **Executive Skills** | Stroop Test  Trails B test  Cantab ID/ED switch |
| **Processing Speed** | Digit Symbol Modality Test (DSMT - mean of oral and written)  Phonemic Verbal Fluency  Semantic Verbal Fluency  Trails A test |
| **Non-verbal Skills/Memory** | Rey-Osterrieth Complex Figure (mean of copy and delays)  WASI Block Design sub-test |

**Table S2: Complete demographic and radiological data for successfully recruited patients**

| **Patient** | **Age** | **Sex** | **Location** | **Side** | **WFNS** | **Fisher** | **Hydrocephalus** |
| --- | --- | --- | --- | --- | --- | --- | --- |
| **1** | 77 | Female | Distal ACA | Left | 1 | 4 | Mild/moderate |
| **2** | 30 | Male | A1/A2 Junction | Left | 1 | 4 | None |
| **3** | 55 | Male | MCA bifurcation | Left | 1 | 4 | None |
| **4** | 47 | Female | Distal ACA | Right | 2 | 4 | None |
| **6** | 41 | Female | MCA bifurcation | Left | 1 | 4 | None |
| **7** | 62 | Female | MCA bifurcation | Left | 2 | 4 | Mild/moderate |
| **8** | 37 | Female | PCommA | Left | 1 | 4 | None |
| **9** | 44 | Female | PICA | Right | 1 | 4 | Mild/moderate |
| **10** | 62 | Male | PCommA | Right | 1 | 4 | None |
| **11** | 58 | Female | PCommA | Right | 1 | 4 | None |
| **12** | 48 | Female | ACommA | Midline | 2 | 4 | Severe |
| **13** | 70 | Female | PCommA | Right | 2 | 4 | Severe |
| **14** | 45 | Male | ICA | Left | 1 | 4 | None |
| **15** | 66 | Female | MCA bifurcation | Right | 1 | 3 | None |
| **16** | 44 | Female | A1/A2 junction | Left | 1 | 3 | None |
| **17** | 43 | Male | Para-opthalmic | Left | 1 | 4 | Mild/moderate |
| **18** | 60 | Male | A1/A2 junction | Right | 2 | 3 | Mild/moderate |
| **19** | 69 | Female | MCA | Left | 1 | 3 | None |
| **20** | 66 | Female | A1/A2 junction | Left | 1 | 4 | Mild/moderate |
| **21** | 69 | Female | PCommA | Right | 2 | 4 | Mild/moderate |
| **22** | 51 | Male | A1/A2 junction | Right | 1 | 4 | Mild/moderate |
| **23** | 59 | Female | PCommA | Left | 2 | 3 | None |
| **24** | 39 | Female | A1/A2 junction | Right | 2 | 4 | Mild/moderate |
| **25** | 67 | Male | MCA bifurcation | Right | 1 | 4 | None |
| **26** | 49 | Male | A1/A2 junction | Right | 1 | 3 | None |
| **27** | 68 | Female | A1/A2 junction | Right | 1 | 3 | Mild/moderate |
| **29** | 51 | Female | ACommA | Midline | 1 | 4 | Mild/moderate |

**ACA** = Anterior communicating artery, **ACommA** = Anterior communicating artery, **PCommA** = Posterior communicating artery, **MCA** = Middle cerebral artery, **ICA** = Internal carotid artery, **A1** = Anterior cerebral artery A1 segment, **A2** = Anterior cerebral artery A2 segment, **PICA** = Posterior, inferior cerebellar artery

**Table S3: Longitudinal MRI analyses within the patient group**

| **SIENA-X** | **Assessment 1 – Assessment 2** | **Assessment 1 – Assessment 3** |
| --- | --- | --- |
| Total brain volume | p=0.62 | p=0.004 |
| Grey matter volume | p=0.54 | p=0.01 |
| White matter volume | p=0.03 | p=0.02 |
| Ventricular CSF volume | p=0.02 | p=0.03 |
|  |  |  |
| **DWI** |  |  |
| Mean grey matter ADC | p=0.22 | p=0.001 |

**Table S4:** Longitudinal GM volume changes in the posterior cerebellum at each MRI scan timepoint for patients with neurocognitive impairment at 3 months and patients with no neurocognitive impairment at 3 months.

**Patients with neurocognitive deficit at 3 months**

| **Patient ID** | **Cerebellum ROI normalised GM volume (a.u.)** | | |
| --- | --- | --- | --- |
|  | **Scan 1** | **Scan 2** | **Scan 3** |
| **1** | 0.184 | 0.163 | 0.153 |
| **2** | 0.205 | 0.198 | 0.195 |
| **3** | 0.190 | 0.166 | 0.166 |
| **4** | 0.187 | 0.184 | 0.176 |
| **5** | 0.205 | 0.205 | 0.203 |
| **6** | 0.194 | 0.178 | 0.165 |
| **7** | 0.166 | 0.171 | 0.176 |
| **8** | 0.158 | 0.136 | 0.147 |
| **9** | 0.193 | 0.164 | 0.145 |
| **10** | 0.183 | 0.171 | 0.162 |
| **11** | 0.214 | 0.187 | 0.182 |

**Patients without neurocognitive deficit at 3 months**

| **Patient** | **Cerebellum ROI normalised GM volume (a.u.)** | | |
| --- | --- | --- | --- |
|  | **Scan 1** | **Scan 2** | **Scan 3** |
| **1** | 0.176 | 0.183 | 0.168 |
| **2** | 0.172 | 0.167 | 0.154 |
| **3** | 0.147 | 0.177 | 0.163 |
| **4** | 0.167 | 0.156 | 0.163 |
| **5** | 0.147 | 0.147 | 0.156 |
| **6** | 0.165 | 0.152 | 0.143 |
| **7** | 0.163 | 0.184 | 0.155 |
| **8** | 0.207 | 0.189 | 0.141 |
| **9** | 0.195 | 0.146 | 0.133 |
| **10** | 0.149 | 0.147 | 0.134 |
| **11** | 0.136 | 0.164 | 0.155 |
| **12** | 0.154 | 0.155 | 0.163 |
| **13** | 0.171 | 0.165 | 0.172 |
| **14 (only completed one scan)** | 0.175 | X | X |

**Figure S1: Box and whisker plots showing data for each neurocognitive test at 3 months following SAH.**

Individual data points for each patient are plotted on top of each index box-and-whisker plot with tests grouped by the cognitive domain assessed. The line in the box plot indicates the median value, the width of the box shows the interquartile range and the whiskers show the minimum and maximum values. The hatched line on the y-axis represents a z-score of -1.67 corresponding to the 5th centile.

**Figure S2: Global (SIENA-X) and regional (VBM) GM volume longitudinal changes between the acute phase (within <72 hours of the SAH) and 3 months post-SAH in the patient group.** **A:** Results from the SIENA-X analysis show a significant decrease in whole brain GM volume at 3 months. **B:** Results from the regional GM (VBM) analysis show widespread apparent decrease in GM volume at 3 months (in blue, p<0.001 TFCE-corrected).

**Figure S3:** Longitudinal GM volume changes in the posterior cerebellum at each MRI scan timepoint for patients with neurocognitive impairment at 3 months (**A)** and patients with no neurocognitive impairment at 3 months (**B).** Each line on the graphs represents a single patient dataset.
